# Functional characterization of the sciarid *BhC4-1 *core promoter in transgenic *Drosophila*

**DOI:** 10.1186/1471-2199-12-32

**Published:** 2011-08-01

**Authors:** Adriana C Garcia, Daniel LG Gitaí, Fernanda C Humann, Maria L Paçó-Larson, Nadia Monesi

**Affiliations:** 1 Departamento de Análises Clínicas, Toxicológicas e Bromatológicas, Faculdade de Ciências Farmacêuticas de Ribeirão Preto, Universidade de São Paulo, Ribeirão Preto, SP, Brazil, 14040-903; 2 Departamento de Biologia Celular, Molecular e de Bioagentes Patogênicos, Faculdade de Medicina de Ribeirão Preto, Universidade de São Paulo, Ribeirão Preto, SP, Brazil, 14049-900; 3 Setor de Biologia Molecular e Genética, Instituto de Ciências Biológicas e da Saúde, Universidade Federal de Alagoas, Maceió, AL, Brazil, 57072-900

## Abstract

**Background:**

Core promoters are *cis*-regulatory modules to which bind the basal transcriptional machinery and which participate in the regulation of transcription initiation. Although core promoters have not been extensively investigated through functional assays in a chromosomal context, the available data suggested that the response of a given core promoter might vary depending on the promoter context. Previous studies suggest that a (-57/+40) fragment constitutes the core promoter of the *BhC4-1 *gene which is located in DNA puff C4 of the sciarid fly *Bradysia hygida*. Here we tested this (-57/+40) fragment in distinct regulatory contexts in order to verify if promoter context affects its core promoter activity.

**Results:**

Consistent with the activity of a core promoter, we showed that in the absence of upstream regulatory sequences the (-57/+40) fragment drives low levels of reporter gene mRNA expression throughout development in transgenic *Drosophila*. By assaying the (-57/+40) fragment in two distinct regulatory contexts, either downstream of the previously characterized *Fbp1 *enhancer or downstream of the UAS element, we showed that the *BhC4-1 *core promoter drives regulated transcription in both the germline and in various tissues throughout development. Furthermore, the use of the *BhC4-1 *core promoter in a UAS construct significantly reduced salivary gland ectopic expression in third instar larvae, which was previously described to occur in the context of the GAL4/UAS system.

**Conclusions:**

Our results from functional analysis in transgenic *Drosophila *show that the *BhC4-1 *core promoter drives gene expression regardless of the promoter context that was assayed. New insights into the functioning of the GAL4/UAS system in *Drosophila *were obtained, indicating that the presence of the SV40 sequence in the 3' UTR of a UAS construct does not preclude expression in the germline. Furthermore, our analysis indicated that ectopic salivary gland expression in the GAL4/UAS system does not depend only on sequences present in the GAL4 construct, but can also be affected by the core promoter sequences in the UAS construct. In this context, we propose that the sciarid *BhC4-1 *core promoter constitutes a valuable core promoter which can be employed in functional assays in insects.

## Background

One of the most important events in the control of gene expression is the regulation of transcription initiation. Transcription of eukaryotic protein-coding genes by RNA polymerase II involves a tightly regulated sequence of steps including decondensation of the *locus*, nucleosome remodeling, histone modifications, binding of transcriptional activators and coactivators to enhancers and promoters that culminates in the recruitment of the basal transcription machinery to the core promoter [1-3]. The basal transcription machinery comprises a set of factors, including RNA polymerase II, that are minimally essential for *in vitro *transcription from an isolated core promoter, whereas the RNA polymerase II core promoter has been defined as a *cis*-regulatory module whose function is to direct the initiation of transcription [2].

Recent studies have revealed that the structure and function of core promoters is more diverse than previously thought. The majority of the eukaryotic core promoters are classified as focused core promoters [1,4]. In a focused core promoter transcription starts at a single nucleotide or within a distinct cluster of start sites in a short region of several nucleotides. Studies in vertebrates have also revealed a second type of core promoter, named dispersed core promoters, in which several weak transcription start sites are distributed over a broad region ranging from 50 to 100 nucleotides [1,4].

Even though there are apparently no universal core promoter elements, molecular studies on focused core promoters have identified and characterized sequence motifs that are characteristic of core promoters such as the Inr (Initiator), TATA box, BRE (TFIIB Recognition Element), DPE (Downstream core Promoter Element), MTE (Motif Ten Element) and DCE (Downstream Core Element) [1,2,4]. Central to the transcription initiation process is the binding of TBP [TATA binding protein, a subunit of TFIID (Transcription Factor IID)] to DNA at a specific distance from the transcription start site, regardless of the presence or absence of a TATA box. The core promoter motifs usually work in cooperation and biochemical studies have demonstrated synergism between different pairs of core promoter motifs [2-4]. These studies of core promoter motifs have been further extended by the use of computational approaches [5-7]. Furthermore, core promoter function is not restricted to the binding of general transcription factors. Several studies have shown the existence of specificity between enhancers and core promoters demonstrating that core promoter motifs constitute *cis*-regulatory elements that participate in transcriptional regulation [8-10] and are an important component in transcriptional networks [11].

Focused core promoters have also been identified in the regulatory region of sciarid DNA puff genes [12-16]. In addition to RNA puffs, which are of general occurrence in Diptera, the polytene chromosomes of sciarid flies also present DNA puffs that are formed at discrete sites in the salivary gland polytene chromosomes at the end the last larval instar. The molecular characterization of DNA puffs revealed that they constitute sites of developmentally regulated gene amplification and transcription [17-19]. These three processes, gene amplification, gene expression and DNA puff formation are all induced in the salivary glands as a late response to the increased ecdysone levels that trigger metamorphosis [20,21].

Earlier studies have shown that the *BhC4-1 *DNA puff gene of the sciarid *Bradysia hygida *is amplified 21-fold and is abundantly transcribed in the salivary gland when DNA puff C4 is formed at the end of the fourth larval instar [13,22]. The characterization of the mechanisms that regulate *BhC4-1 *expression have been extended through functional studies performed in transgenic *Drosophila*, and have resulted in the identification of *cis*-regulatory elements in the *BhC4-1 *promoter region [23-25]. Similar to events in the sciarid, in transgenic *Drosophila BhC4-1 *expression in the salivary gland is induced as a late response to the increase in ecdysone levels [20]. In addition, genetic interaction experiments have shown that the *BhC4-1 cis*-regulatory elements are recognized by *trans*-activating factors of *D. melanogaster*, indicating that the regulatory mechanisms of *BhC4-1 *transcription in the salivary gland are highly conserved in *D. melanogaster *[26].

Functional studies performed in the *BhC4-1 *promoter region resulted in the identification of a 97 bp fragment (-57/+40) that could not drive detectable levels of β-galactosidase activity and that contained sequences similar to core promoter elements [24]. In this paper, we initially demonstrate that the 97 bp (-57/+40) *BhC4-1 *promoter fragment drives low levels of *BhC4-1-lacZ *mRNA expression throughout development. The few studies available that have investigated core promoters through functional assays in a chromosomal context have shown that the response of a given core promoter can vary depending on the enhancer that is being tested [8-10]. In this context, we investigated the 97 bp (-57/+40) *BhC4-1 *core promoter fragment in two distinct regulatory frameworks: downstream of the previously characterized *D. melanogaster Fbp1 *(*Fat body protein 1*) enhancer [27] and downstream of 5 copies of the UAS element, in the context of the GAL4/UAS system [28]. Our results reveal that the *BhC4-1 *core promoter drives regulated transcription in the germline and in various tissues at different developmental stages, regardless of the regulatory framework that is being tested. Additionally, our results provide novel insights regarding the functioning of the GAL4/UAS system in both the germline and in the larval salivary gland of *D. melanogaster*.

## Results and discussion

### The *BhC4-1 *core promoter drives basal levels of *BhC4-1-lacZ *expression throughout development

**Figure 1:**
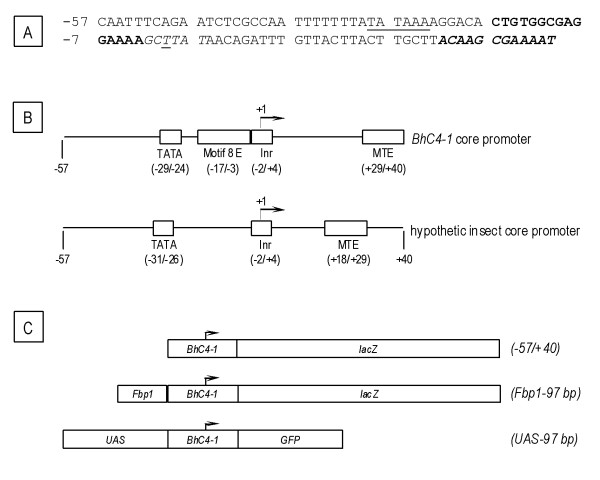
**The *BhC4-1 *core promoter and diagrams of the analyzed constructs**. The sequence of the 97 bp (-57/+40) *BhC4-1 *core promoter region [GenBank:U13892] is shown in (A). The TATA motif and the initiator element (Inr) are underlined and in *italic*, respectively. The experimentally defined transcription start site (+1) is the first T (*underlined, italic*) in the Inr element [13]. The sequences similar to Motif 8 and Motif 10 (MTE) [7,29] are shown in *bold *and *bold/italic*, respectively. (B) The upper diagram shows the core promoter elements identified in the *BhC4-1 *core promoter. The numbers below the diagram correspond to the position of each element, defined in relation to the experimentally mapped transcription start site (+1) [13]. The lower diagram, drawn for comparison, consists of a hypothetical insect core promoter containing a TATA motif, and Inr and an MTE. The numbers below the diagram are defined in relation to the transcription start site (+1), and indicate the position in which these elements are more commonly found in insect core promoters. The core promoter elements drawn in the lower diagram consist of the subset of core promoter elements that have been identified. A particular core promoter might contain some, all or none of these elements [2,4]. The motif 8 element (Motif 8 E) is not shown in the bottom diagram because its preferential location in insect core promoters has not been defined. (C) Diagrams of the transgenes analyzed in this work. The arrow above the *BhC4-1 *core promoter diagram indicates the transcription start site and the direction of transcription.

Previous studies from our group have suggested that a 97 bp (-57/+40) fragment contained the *BhC4-1 *core promoter [24]. This suggestion was derived both from functional assays demonstrating that lines transformed with a *(-57/+40) *construct did not present detectable levels of β-galactosidase expression, and from sequence analysis showing the presence of a TATA box located 28 bp upstream from the initiator element (Inr) in the (-57/+40) fragment (Figure 1A, B) [13,24].

Here we have extended the analysis of the (-57/+40) sequence and identified two additional motifs that are prevalent in insect core promoters [7]. A sequence similar (9/15) to the motif 8 element (MKSYGGCARCGSYSS) is found between positions (-17/-3) and a sequence similar (7/12) to the motif ten element (MTE) (CSARCSSAACGS) is found between positions (+29/+40) (Figure 1A, B). The motif 8 element does not seem to have a preferential localization in core promoters and, at present, the transcription factors that bind to this motif have not been identified [7]. The motif ten element (MTE) is bound by the TFIID complex, is localized downstream of the Inr element between positions (+18/+29), and there is a strict spacing requirement between the Inr and the MTE (Figure 1B) [29]. In the *BhC4-1 *core promoter the sequence similar to the MTE is found further downstream of the transcription start site (+29/+40) (Figure 1B), and hence this sequence most likely does not contribute to the binding of the basal transcriptional machinery.

**Figure 2:**
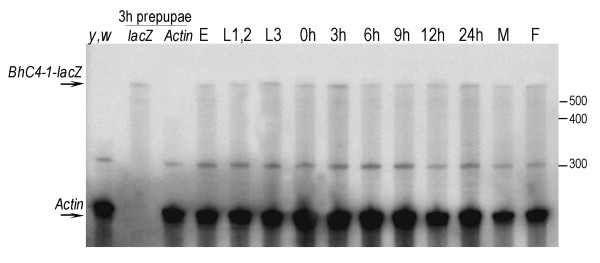
***BhC4-1-lacZ *mRNA expression in a *(-57/+40) *transgenic line**. Thirty micrograms of total RNA extracted from embryos (E), first and second instar larvae (L1,2), third instar larvae (L3), prepupae at 0 h, 3 h, 6 h, 9 h and 12 h, 24 h pupae, adult males (M) and adult females (F) were analyzed by the Ribonuclease Protection Assay using a mixture of two radiolabeled probes. The protected *BhC4-1-lacZ *RNA mRNA is 617 nt long (*BhC4-1-lacZ *arrow). The protected *Actin *mRNA is about 260 nt long (*Actin *arrow). In lane (*y,w*), 30 μg of total RNA extracted from embryos of the parental strain *y,w *were hybridized with both probes. In lanes (3 h prepupae/*lacZ*) and (3 h prepupae/*Actin*), 30 μg of total RNA extracted from 3 h prepupae of the *(-57/+40) *transgenic line were hybridized either with the *lacZ *probe or the *Actin *probe, as indicated. The migration of the RNA size markers is indicated on the right.

As explained above, lines transformed with the *(-57/+40) *construct did not present detectable levels of β-galactosidase expression. In order to verify if the lack of β-galactosidase expression in lines transformed with the *(-57/+40) *construct (Figure 1C) [24] was due to the absence of *BhC4-1-lacZ *mRNA transcription, we performed RNase Protection Assays in two independent lines transformed with the *(-57/+40) *construct. In both transgenic lines, low levels of *BhC4-1-lacZ *mRNA expression were detected at all developmental stages analyzed (Figure 2; Additional file 1). *BhC4-1-lacZ *mRNA expression is not detected in the control, total RNA extracted from embryos of the parental strain, *y,w*, (Figure 2; Additional file 1; lanes *y,w*), indicating that there is no endogenous *BhC4-1-lacZ *mRNA expression in the parental strain. The pattern of *BhC4-1-lacZ *mRNA expression in the two independent transgenic lines is similar, indicating that it constitutes the pattern of expression of the transgene and is not the result of position effects. The suggestion that the detected *BhC4-1-lacZ *mRNA is a transcription product driven by the *BhC4-1 *core promoter is further supported by the fact that the vector employed to build the *(-57/+40) *construct, pCaSpeR-AUG-βgal does not contain promoter sequences upstream of the *lacZ *reporter gene [30]. In this context, we conclude that the 97 bp (-57/+40) *BhC4-1 *core promoter drives low levels of transcription throughout development, and attribute the absence of detectable levels of β-galactosidase activity in these lines to the expression of low levels of *BhC4-1-lacZ *mRNA in the *(-57/+40) *lines.

The components of the basal transcription machinery that bind to the 97 bp (-57/+40) *BhC4-1 *promoter fragment have not yet been identified. However, since in transgenesis the 97 bp (-57/+40) fragment drives low levels of *BhC4-1-lacZ *mRNA transcription throughout development, and the spacing between the TATA box and the Inr is compatible with the spacing described for these motifs in core promoters [2,4,7], we propose that these two motifs are relevant for the function of the *BhC4-1 *core promoter.

### The interaction between the sciarid *BhC4-1 *core promoter and the drosophilid *Fbp1 *enhancer results in enhancer-dependent transgene expression

**Figure 3:**
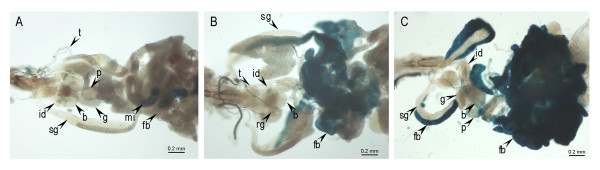
**β-galactosidase expression in the fat body of larvae and prepupae of the *Fbp1-97bp *transgenic line**. (A) 96 h third instar larvae, (B) 100 h third instar larvae and (C) 0 h prepupae. Reporter gene expression was detected after 1 h incubation in the presence of X-gal. The arrows indicate the trachea (t), proventriculus (p), imaginal discs (id), salivary glands (sg), brain (b), ventral ganglion (g), midintestine (mi), fat body (fb) and ring gland (rg), respectively. The blue staining detected in the imaginal discs (id) and in the midintestine (mi) corresponds to endogenous β-galactosidase activity [56].

Functional studies of the *BhC4-1 *promoter have identified a salivary gland enhancer (-186/-58) that drives gene expression in prepupal salivary glands, and a ring gland enhancer (-253/-187) that promotes expression in the ring gland of late embryos and throughout the larval and prepupal stages [24,25]. In order to verify if the sciarid *BhC4-1 *core promoter could drive expression in another tissue when placed downstream of a heterologous tissue specific enhancer, we generated transgenic lines transformed with the *Fbp1-97 bp *construct (Figure 1C). This construct contains a 70 bp (-138/-69) fat body enhancer from the *D. melanogaster Fbp1 *gene [27] which was cloned upstream the 97 bp (-57/+40) *BhC4-1 *core promoter in the pCaSpeR-AUG-β-gal vector [30]. β-galactosidase activity was assayed in nine independent transgenic lines and representative results obtained for one of the lines are shown in Figure 3. Analysis of staged animals of the *Fbp1-97 bp *lines revealed that reporter gene expression in the fat body starts between 96 h to 100 h after egg deposition and continues to be detected in this tissue throughout the prepupal and pupal stages (Figure 3 and data not shown). In *Drosophila, Fbp1 *expression is restricted to the fat body, starts in third instar larvae before the onset of wandering, continues during the prepupal stage and decreases at the beginning of the pupal stage [31-33]. In this context, we conclude that the pattern of expression of the *Fbp1-97 bp *transgene is similar to the pattern of expression of the endogenous *Fbp1 *gene.

In lines transformed with a construct containing the (-138/-69) *Fbp1 *enhancer cloned upstream the *hsp70 *core promoter, reporter gene expression was consistently detected both in the fat body and in the anterior region of the salivary gland [27]. In the *Fbp1-97 bp *lines, reporter gene activity was restricted to the fat body. It is possible that the *BhC4-1 *core promoter presents tissue specific differences in activity that precludes its activation in the salivary gland.

When either the *BhC4-1 *salivary gland enhancer or the *BhC4-1 *ring gland enhancer were tested upstream of the (-60/+80) *Fbp1 *core promoter, high levels of developmentally regulated reporter gene expression were observed [25], indicating that the enhancers and core promoters of both *BhC4-1 *and *Fbp1 *genes are interchangeable. One possible explanation for this interchangeability is the fact that both are ecdysone responsive genes that are expressed in a developmentally regulated manner in specific tissues [20,33-36]. In summary, we conclude that the developmentally regulated pattern of reporter gene expression observed in the fat body of the *Fbp1-97 bp *lines is driven by the interaction between the *BhC4-1 *sciarid core promoter and the *Fbp1 *fat body enhancer of *D. melanogaster*.

### The *BhC4-1 *core promoter drives regulated transcription in the context of the GAL4/UAS system

**Figure 4:**
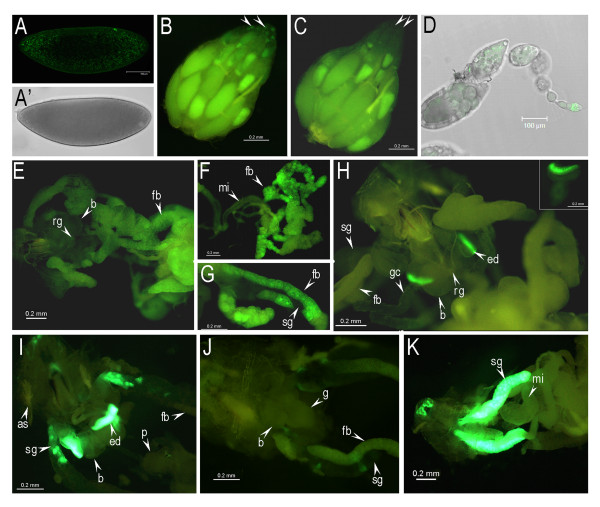
**Patterns of GFP expression driven by different GAL4 lines**. (A) *UAS-97 bp/GAL4-twi.2xPE *3-4 h old embryo; (B) whole ovary dissected from an *UAS-97 bp; GAL4::VP16-nos.UTR *female. The arrows indicate the germarium; (C) whole ovary from an *UAS-97 bp *female, note the absence of GFP expression at the tip of the ovary where the germarium is located (arrows); (D) dissected ovariole from an *UAS-97 bp; GAL4::VP16-nos.UTR *female; (E) dissected *UAS-97 bp; Lsp2-GAL4 *third instar larvae showing GFP expression in the larval fat body; (F, G) dissected fat body and associated organs from an *UAS-97 bp; Lsp2-GAL4 *early prepupae, note that GFP expression is only verified in the fat body; (H) dissected *UAS-97 bp/GAL4-GMR *larva, the inset shows a dissected antenna-eye imaginal disc from a larva of the same genotype, note that GFP expression occurs only in the eye imaginal disc; (I) dissected *UAS-GFP.S65T/GAL4-GMR *larva showing GFP expression both in the eye disc and in the salivary gland; (J) dissected *UAS-97 bp; pPTGAL26 *larva, note the presence of a low frequency of GFP positive cells in the salivary glands; (K) dissected *UAS-GFP.S65T; pPTGAL26 *larva, note GFP expression in the salivary glands. (A) confocal microscopy; (A') bright field image of the same embryo as shown in (A), (B, C, E, F, G, H, I, J, K) fluorescence stereoscopy; (D) overlay between confocal microscopy and bright field. (rg) ring gland, (b) brain, (fb) fat body, (mi) midintestine, (sg) salivary gland, (gc) gastric caeca, (ed) eye imaginal disc, (as) anterior spiracle, (p) proventriculus, (g) ventral ganglion.

The GAL4/UAS system has been extensively employed to drive tissue-specific expression of genes of interest in *Drosophila *[28,37]. In order to test whether the (-57/+40) *BhC4-1 *core promoter could drive efficient transcription in the context of the GAL4/UAS system, we exchanged the *hsp70 *core promoter for the (-57/+40) *BhC4-1 *core promoter in the pUAST vector [28]. The *GFP *coding sequence was cloned downstream of the *BhC4-1 *core promoter and the resulting construct, *UAS-97 bp*, (Figure 1C) was inserted into *Drosophila *by germline transformation. The resulting four transgenic lines did not present GFP expression in any developmental stage and/or tissue, as verified by fluorescence stereoscopy and microscopy (Figure 4C; Additional file 2A, B, F, G and data not shown).

In order to investigate if the *BhC4-1 *core promoter could drive gene expression during embryogenesis, we crossed the *UAS-97 bp *lines with the *GAL4-twi.2xPE *driver. In this line, GAL4 expression in the ventral region of early embryos is driven by two copies of the proximal element (PE), previously characterized in the promoter region of the *twist *gene [38]. In early *UAS-97 bp/GAL4-twi.2xPE *embryos GFP expression is detected in the ventral region of early embryos and also in the posterior and anterior regions of the embryo (Figure 4A). Since in *UAS-97 bp/GAL4-twi.2xPE *embryos GFP expression was not restricted to the ventral region, we crossed the *GAL4-twi.2xPE *line with the *UAS-GFP.S65T *line, a *UAS-GFP *line that has been previously used in studies that employed the GAL4/UAS system [39-41]. A similar pattern of GFP expression was observed in the *UAS-GFP.S65T/GAL4-twi.2xPE *embryos (Additional file 3). Together these results reveal that the *BhC4-1 *core promoter drives transcription during embryogenesis in the context of the GAL4/UAS system and in a pattern analogous to that observed when a previously characterized UAS line is employed.

In order to investigate whether the *BhC4-1 *core promoter could drive expression in the germline, we crossed the *UAS-97 bp *lines with the *GAL4::VP16-nos.UTR *line that expresses GAL4 in the germ cells [42]. In whole ovaries of *UAS-97 bp; GAL4::VP16-nos.UTR *adult females, GFP expression is verified in the germarium that is localized at the tip of the ovaries (Figure 4B), whereas GFP expression is not detectable in the germarium of ovaries of control *UAS-97 bp *adult females (Figure 4C). When dissected *UAS-97 bp; GAL4::VP16-nos.UTR *ovaries were analyzed using confocal microscopy, we also detected GFP expression in later stages of oogenesis (Figure 4D). We attributed the fluorescence observed in later stages of oogenesis and detected in the ovaries of both *UAS-97 bp; GAL4::VP16-nos.UTR *and *UAS-97 bp *adults to autofluorescence of the yolk (Figures 4B, C). The autofluorescence from the yolk, with a wavelength in the 560 nm - 590 nm range, was not detected when the ovaries were analyzed in the confocal microscope configured to capture only the fluorescence emitted by GFP (from 500 nm to 535 nm). Together, these results show that in *UAS-97 bp; GAL4::VP16-nos.UTR *ovaries GFP is expressed in a pattern similar to that described for the *nanos *transcript [43].

One limitation of the original GAL4/UAS system was the inability to work in the germline during oogenesis [28]. This restriction was overcome with utilization of the UASp vector in which the *D. melanogaster hsp70 *core promoter, part of the original pUAST vector, was substituted by the minimal promoter and first intron of the *D. melanogaster P transposase *gene [44]. In the UASp vector, the 3' end SV40 sequence of pUAST was substituted by the 3' UTR of *K10*, and it has been suggested that the ability of a UAS construct to work in the germline depended on both the core promoter and on the 3' sequences [44]. The *UAS-97 bp *construct contains the 3' end SV40 sequence which is part of the original pUAST vector [28]. At present we cannot rule out the possibility that the 3' end SV40 sequence might affect the *P transposase *promoter in the context of the UASp vector. However, since GFP expression was detected in ovaries of *UAS-97 bp; GAL4::VP16-nos.UTR *females (Figures 4B, D), we suggest that the 3' UTR sequences tested to date (SV40 and *K10*) most likely do not interfere with the transactivation of a UAS construct in the germline.

We next crossed the *UAS-97 bp *lines with two drivers that express GAL4 in two distinct larval tissues. The *Lsp2-GAL4 *line expresses GAL4 in the larval, prepupal and pupal fat body according to the pattern of expression of the *Lsp2 *gene [32]. The *GAL4-GMR *line expresses GAL4 in all cells posterior to the morphogenetic furrow [45]. Fluorescence stereoscopy of third instar larvae revealed GFP expression both in the fat body of *UAS-97 bp; Lsp2-GAL4 *animals (Figure 4E, F, G, Additional file 2I) and in the posterior region of eye imaginal discs of *UAS-97 bp/GAL4-GMR *larvae (Figure 4H and inset). In both cases GFP expression was clearly differentiated from the autofluorescence present in larvae of either the parental line, *yw *(Additional file 2C), or the *UAS-97 bp *lines (Additional file 2A, B, G) or the *Lsp2-GAL4 *line (Additional file 2D, H) or the *GAL4-GMR *lines (data not shown), showing that the observed patterns of GFP expression were driven by the respective GAL4 drivers.

These results revealed that in the context of the GAL4/UAS system the *BhC4-1 *core promoter drives regulated transcription in developmental times (embryogenesis, adults) and tissues (ovaries, imaginal discs and fat body) other than those in which the *BhC4-1 *enhancers have been shown to drive reporter gene expression, namely the ring gland of late embryos, larvae and prepupae and the prepupal salivary glands [24,25]. The results verified in both the *UAS-97 bp *lines and in the *Fbp1-97 bp *lines agree with those previously obtained with the *(-253/+40) *and *(-186/+40) *lines [24], and indicate that the regulated expression driven by the (-57/+40) *BhC4-1 *core promoter in transgenic *Drosophila *depends on the regulatory sequences placed upstream the (-57/+40) fragment. Our data are in contrast with those recently reported in *Tribolium castaneum *showing that transactivation of the GAL4/UAS system was only achieved when an endogenous core promoter was employed in both the driver and responder constructs [46]. One possibility is that *D. melanogaster *is more permissive towards the use of a heterologous core promoter. A further possibility is that because of the greater evolutionary distance between the Dipteran *Drosophila *and the Coleopteran *Tribolium*, the proteins and DNA sequences involved in promoter recognition have diverged more than between the two Dipteran *Drosophila *and *Bradysia*. In this sense, we suggest that the *BhC4-1 *core promoter might constitute an attractive alternative core promoter to be employed in other insect systems that are amenable to transformation.

### The presence of the *BhC4-1 *core promoter in a UAS construct reduces the levels of ectopic salivary gland expression that are intrinsic to the GAL4/UAS system

As explained in the previous section, similar patterns of expression were observed in embryos when the same GAL4 driver, *GAL4-twi.2xPE*, was crossed either with the *UAS-97 bp *lines or with the previously employed *UAS-GFP.S65T *line (Figure 4A, Additional file 3). A distinct result was obtained when we compared the pattern of GFP expression in *UAS-97 bp/GAL4-GMR *larvae (Figure 4H) with the pattern of GFP expression in *UAS-GFP.S65T/GAL4-GMR *larvae (Figure 4I). In the *UAS-GFP.S65T/GAL4-GMR *larvae, GFP expression was detected both in the eye imaginal discs and in the salivary glands (Figure 4I), whereas in the *UAS-97 bp/GAL4-GMR *larvae GFP expression was only observed in the eye imaginal discs (Figure 4H). To investigate if the difference between these two transactivation patterns could be due to variations in the UAS lines, we crossed a second GAL4 line, *pPTGAL26*, with either the *UAS-97 bp *line or with the *UAS-GFP.S65T *line. The transgenic line *pPTGAL26 *contains one copy of the pPTGAL vector [47] and expresses GAL4 in the salivary gland. In *UAS-GFP.S65T; pPTGAL26 *larvae, strong GFP expression was observed in the larval salivary glands (Figure 4K), whereas in *UAS-97 bp; pPTGAL26 *larvae GFP expression is barely detected in the salivary glands (Figure 4J). Together, these results reveal that distinct patterns of transactivation can be produced in larvae when a given GAL4 driver is crossed with two distinct UAS lines.

The differences observed in GFP expression in the salivary gland when the two different UAS lines were employed cannot be attributed to the GFP variant being used because both constructs contain the S65T variant [48], nor to differences in the 3' UTR because both constructs contain the 3' UTR SV40 sequence of the original pUAST vector [28]. Furthermore, since similar results were obtained when independent *UAS-97 bp *lines were crossed with the same GAL4 drivers (data not shown), we could rule out the contribution of position effects.

We propose that the observed differences in ectopic GFP expression in the salivary gland of larvae when either UAS line was employed can be attributed to several factors. The results with the *pPTGAL *lines (Figure 4J, K) confirm that ectopic salivary gland expression in the context of the GAL4/UAS system can be attributed to the GAL4 component of the GAL4/UAS system, as previously suggested when the GAL4/UAS system in *D. melanogaster *was originally described [28]. In addition, our results indicate that the UAS component also contributes to ectopic salivary gland expression in the context of the GAL4/UAS system. Specifically, we suggest that the substitution of the *D. melanogaster hsp70 *core promoter present in *UAS-GFP.S65T *construct by the sciarid *BhC4-1 *core promoter in the *UAS-97 bp *construct led to a strong reduction in salivary gland ectopic expression in animals containing the *UAS-97 bp *construct, irrespective of the driver line being employed (Figure 4H, J). The possibility that these differences in core promoter activity might be due to the presence of *cis*-regulatory elements that drive salivary gland expression in the *hsp70 *core promoter is supported by the observation of GFP expression in third instar larvae salivary glands of the *UAS-GFP.S65T *line in the absence of a GAL4 driver (Additional file 2E, F). It is possible that the *BhC4-1 *core promoter does not contain *cis*-regulatory elements that drive salivary gland expression. Alternatively, these *cis*-regulatory elements might also be present in the *BhC4-1 *core promoter but are specifically repressed in this tissue either due to the presence of tissue specific repressor or due to the absence of required co-activators. Even though at present we are not able to distinguish between these possibilities, we propose that in some situations, when ectopic transactivation in the salivary glands constitutes a problem, the *UAS-97 bp *lines generated in this work might be employed as a useful alternative to the widely used *UAS-GFP.S65T *line.

## Conclusions

In the absence of upstream activating sequences the (-57/+40) *BhC4-1 *core promoter drives low levels of expression throughout development in transgenic *Drosophila *which is consistent with the function of a core promoter. When placed downstream of different regulatory elements from either *B. hygida, D. melanogaster *or yeast the *BhC4-1 *core promoter drives efficient regulated transcription in *Drosophila *in distinct tissues throughout development. In the context of the GAL4/UAS system the *BhC4-1 *core promoter drives gene expression in the germline indicating that the presence of the SV40 sequence in the 3' UTR of a UAS construct does not preclude expression in the germline. In addition, the data derived from the functional characterization of the *BhC4-1 *promoter revealed that ectopic salivary gland expression in the GAL4/UAS system is not solely related to sequences present in the GAL4 construct, but can also be affected by the use of different core promoter sequences in the UAS construct. Our results contribute to the functional characterization of the sciarid *BhC4-1 *core promoter that is an attractive alternative core promoter which might be employed in functional assays in insects.

## Methods

### *Drosophila *transgenic lines, fly rearing and staging

The *(-57/+40) *transgenic lines have been previously described [24]. Briefly, the *(-57/+40) *construct consists of the 97 bp (-57/+40) *BhC4-1 *fragment cloned upstream the *lacZ *reporter gene into the pCaSpeR-AUG-β-gal vector, that does not contain promoter sequences upstream the reporter gene [30]. The line *w[1118]; P{w[+mC] = GAL4::VP16-nos.UTR}MVD1 *(stock # 4937), referred in the text as *GAL4::VP16-nos.UTR*, and the line y[1] w[*]; P{w[+mC] = GAL4-twi.2xPE}2 (stock # 2517), referred in the text as *GAL4-twi.2xPE*, were both obtained from the Bloomington Stock Center. The line w[*]; P{w[+mC] = GAL4-ninaE.GMR}(Bloomington stock # 1104), named *GAL4-GMR *in the text, and the line w[*]; P{w[+mC] = UAS-GFP.S65T}T2 (Bloomington stock # 1521), named *UAS-GFP.S65T *in the text, were kindly provided by Dr. R. G. P. Ramos (FMRP-University of São Paulo). Line *y[1] w [1118]; P{w[+mC] = Lsp2-GAL4.H}3 *(Bloomington stock # 6357), referred in the text as *Lsp2-GAL4*, was provided by Dr. M. L. Paçó-Larson's laboratory. Line *pPTGAL26 *consists of a transgenic line transformed with the pPTGal vector [47]. Salivary gland GAL4 expression was observed in all seven independent lines transformed with the pPTGal vector (M.L. Paçó-Larson and F.C. Humann, unpublished results). *Drosophila *lines were reared at 25°C in standard *Drosophila *medium [49]. Staging of third instar larvae was achieved by rearing the stocks in *Drosophila *medium containing 0.05% bromophenol blue [50].

### Constructs

*Fbp1-97 bp*. The pBC4 57 construct [24] consisted of the 97 bp (-57/+40) *BhC4-1 *core promoter cloned into the Bluescript II KS+ vector (Stratagene). Initially, the pBC4 57 construct was digested with *Eco *RV, followed by dephosphorylation with calf intestinal phosphatase (New England Biolabs). Two complementary oligonucleotides, comprising the 70 bp (-138/-69) *Fbp1 *enhancer [27], were annealed, phosphorylated with polynucleotide kinase (New England Biolabs), filled in with Klenow (New England Biolabs) and re-annealed. The 70 bp *Fbp1 *insert was ligated to the linearized pBC4 57. One of the constructs, containing the *Fbp1 *70 bp enhancer cloned in the correct orientation (Fbp1-ori1) upstream the 97 bp *BhC4-1 *core promoter, was digested with *Xho *I, filled in, digested with *Bam *HI and gel purified, which resulted in a 167 bp fragment. The pCaSpeR-AUG-β-gal vector [30] was digested with *Eco *RI, filled in, digested with *Bam *HI, gel purified and ligated to the 167 bp insert, which resulted in the *Fbp1-97 bp *transgene. The integrity of the obtained constructs was verified both through digestion analyzes and sequencing.

*UAS-97 bp*. This construct is derived from the pUAST vector [28]. A 156 bp fragment containing 5 copies of the UAS element (5XUAS) was amplified employing pUAST as a template and the pUAST Cla and PUAST Hae-Sal primers. By using PUAST Cla (5' CATGAGCTCGGATCGATGCTTG 3') and PUAST Hae-Sal (5' TACTCCGGCCGTCGACAGAGTC 3'), a *Cla *I restriction site was inserted at the 5' end of the 5XUAS fragment and both *Hae *III and *Sal *I restriction sites were inserted at the 3' end of the 5XUAS fragment, respectively. The 156 bp 5XUAS fragment was gel purified and digested with both *Cla *I and *Hae *III. The pBC4 57 construct, containing the 97 bp (-57/+40) *BhC4-1 *core promoter fragment was digested with *Eco *RI, followed by a filled in reaction and a second digestion with *Cla *I and ligated to the 156 bp 5XUAS fragment. The resulting construct (pBS-5XUAS-97 bp) was digested with *Bam *HI, followed by a fill-in reaction, a second digestion with *Sph *I and gel purification of the 219 bp insert (5XUAS-97bp). In parallel the pUAST vector was digested with *Eco *RI, filled in and digested with *Sph *I, which excised a 378 bp fragment containing both the 5XUAS and the *hsp70 *promoter from the pUAST vector. The (5XUAS-97 bp) fragment was ligated to the linearized pUAST. The resulting construct (pUAST-C4) contains the 97 bp (-57/+40) *BhC4-1 *promoter fragment cloned downstream of the 5XUAS element in the pUAST vector. The final step promoted the insertion of the *GFP *sequence downstream of the *BhC4-1 *97 bp promoter. The cdsGFP-PBKS+ contains the *GFP *coding sequence and was digested with *Bam *HI, filled in and digested with *Xba *I. The 715 bp *GFP *coding sequence was ligated to the pUAST-C4 construct that had been previously digested with *Xho *I, filled in, followed by an *Xba *I digestion. The resulting transgene, *UAS-97 bp*, and all intermediate constructs were verified through digestion analyzes and nucleotide sequencing.

### *P*-element transformation

The constructs were injected into embryos of the *y,w *strain of *D. melanogaster *together with the helper plasmid phsπ [51], according to standard protocols [52,53]. Final DNA concentrations were 0,5 μg/ml for the constructs and 0,1 μg/ml for the helper plasmid. The constructs carried the *white *gene which was employed to select the transgenic flies. Surviving G0 flies were individually crossed with the parental strain and G1 independent transgenic flies were employed to establish homozygous and/or balanced lines. Southern blot analysis was performed in all transgenic lines to confirm the integrity of the transformed plasmid, the occurrence of independent transformation events and the number of copies of the construct present in each line.

### Total RNA Extraction

Whole animals at defined developmental stages were collected, frozen in liquid nitrogen and kept at -80°C until further processing. The samples were initially homogenized in lyses buffer (10 mM Tris-HCl pH 9.0; 2% SDS; 50 mM EDTA; 5% ethanol). Total RNA was extracted by adding 10 volumes of Trizol LS (Invitrogen), following the manufacturer's instructions. Total RNA concentrations were estimated by absorbance at 260 nm.

### Ribonuclease Protection Assays

RPAs were performed using the RPA II kit (Ambion), following the manufacturer's instructions. The *lacZ *probe was a 790 bp transcript complementary to 617 nt of the *lacZ *mRNA. The *Actin *probe consisted of a 650 bp *Bgl *II-*Xho *I fragment containing noncoding and coding sequences of the *Drosophila Actin *gene [54], complementary to 260 nt of the *Actin *mRNA. Probes were gel purified before being employed in the assay. The protected fragments were electrophoresed in 4% acrylamide, 8 M urea gels. The gels were dried and directly exposed to PhosphoImager screens (Molecular Dynamics).

### Reporter gene expression analyses

Beta-galactosidase expression was assayed by histochemistry in dissected larvae as previously described [55]. Analyses were performed in a stereoscope MZ12_5 _(Leica), coupled to a digital camera (DC300F), followed by capture and processing using the IM 1000 software (Leica). GFP expression was analyzed in fresh specimens, employing a MZ16F fluorescence stereoscope (Leica), equipped with the fluorescence filter set GFP2 for MZ16 FA, coupled to a digital camera (DC300F), followed by capture and processing using the IM 1000 software (Leica). Confocal microscopy was performed in a Leica TCS-SP2 confocal microscope using the LCS (Leica Confocal Software) (Laboratório de Microscopia Confocal da Faculdade de Medicina de Ribeirão Preto, USP, Brazil).

## Authors' contributions

ACG generated the *UAS-97 bp *lines and analyzed the transactivation patterns in the context of the GAL4/UAS system. DLGG performed the RPAs in the *(-57/+40) *lines and generated and characterized the *Fbp1-97 bp *lines. FCH generated and characterized the *pPTGAL *lines. MLPL participated in the coordination of the study and helped to draft the manuscript. NM conceived the study, did the sequence alignment, participated in the analysis of the transactivation patterns performed in the GAL4/UAS system and drafted the manuscript. All authors read and approved the manuscript.

## Supplementary Material

Additional file 1 ***BhC4-1-lacZ *mRNA expression in an independent *(-57/+40) *transgenic line**. Thirty micrograms of total RNA extracted from embryos (E), first and second instar larvae (L1,2), third instar larvae (L3), prepupae at 0 h, 3 h, 6 h, 9 h and 12 h, 24 h pupae, adult males (M) and adult females (F) were analyzed by the Ribonuclease Protection Assay using a mixture of two radiolabeled probes. The protected *BhC4-1-lacZ *RNA mRNA is 617 nt long (*BhC4-1-lacZ *arrow). The protected *Actin *mRNA is about 260 nt long (*Actin *arrow). In lane (*y,w*), 30 μg of total RNA extracted from embryos of the parental strain *y,w *were hybridized with both probes. In lanes (3 h prepupae/*lacZ*) and (3 h prepupae/*Actin*), 30 μg of total RNA extracted from 3 h prepupae of the *(-57/+40) *transgenic line were hybridized either with the *lacZ *probe or the *Actin *probe, as indicated. The migration of the RNA size markers is indicated on the right. Click here for file

Additional file 2 **Autofluorescence and GFP patterns of expression in control larvae**. Third instar larvae were dissected and images were captured using a fluorescence stereoscope equipped with a GFP optical filter set. All images were captured with similar settings and exposure times. (A), (B) and (G) dissected third instar larvae of independent *UAS-97 bp *transgenic lines. (C) dissected third instar larva of the parental line, *y,w*. (D) and (H) dissected larvae of the *Lsp2-GAL4 *line. The larval tissues are identifiable due to the autofluorescence (yellowish) present in third instar larvae tissues. Note that some tissues (eg. fat body) present higher levels of autofluorescence when compared to others (eg. salivary glands). (E) dissected *UAS-GFP.S65T *third instar larvae. Note the occurrence of GFP expression in the salivary glands and in some groups of cells in the ventral ganglion (F) Salivary glands pairs were dissected either from a *UAS-97 bp *larva (top pair of salivary glands) or from a *UAS-GFP.S65T *larva (bottom pair of salivary glands). Both pairs of salivary glands were imaged together. Note the presence of GFP expression in the *UAS-GFP.S65T *salivary glands. The fat body associated to both pairs of salivary glands is identifiable due to the autofluorescence present in this tissue. The dashed white lines were drawn in order to indicate the location of the *UAS-97 bp *salivary glands in the image field. (I) Dissected *UAS-97 bp; Lsp2-GAL4 *larva. For comparison only the most anterior part of the larvae are shown in (G), (H) and (I). The larva shown in (I) was obtained after crossing the *UAS-97 bp *line (G), with the *Lsp2-GAL4 *line (H). GFP expression (bright green fluorescence) is only observed in the fat body of the *UAS-97 bp; Lsp2-GAL4 *larva (I). (as) anterior spiracle, (fb) fat body, (sg) salivary gland, (g) ventral ganglion, (gc) gastric caeca, (p) proventriculus, (b) brain, (t) trachea, (mi) midintestine, (ids) imaginal discs. Bar, 200 μm. Click here for file

Additional file 3 **Pattern of GFP expression in *UAS-GFP.S65T/GAL4-twi.2xPE *3-4 h old embryo**. Confocal microscopy of an *UAS-GFP.S65T/GAL4-twi.2xPE *3-4 h old embryo. Note that the pattern of GFP expression is similar to the one obtained when the *UAS-97 bp *line was crossed to the same GAL4 driver line (shown in Figure 4A). Click here for file
