# Supplementary figures and images for: Functional characterization of the sciarid BhC4-1 core promoter in transgenic Drosophila
Source: BMC Mol Biol. 2011 Aug 1;12:32. doi: 10.1186/1471-2199-12-32 (PMC3160885; doi:10.1186/1471-2199-12-32)

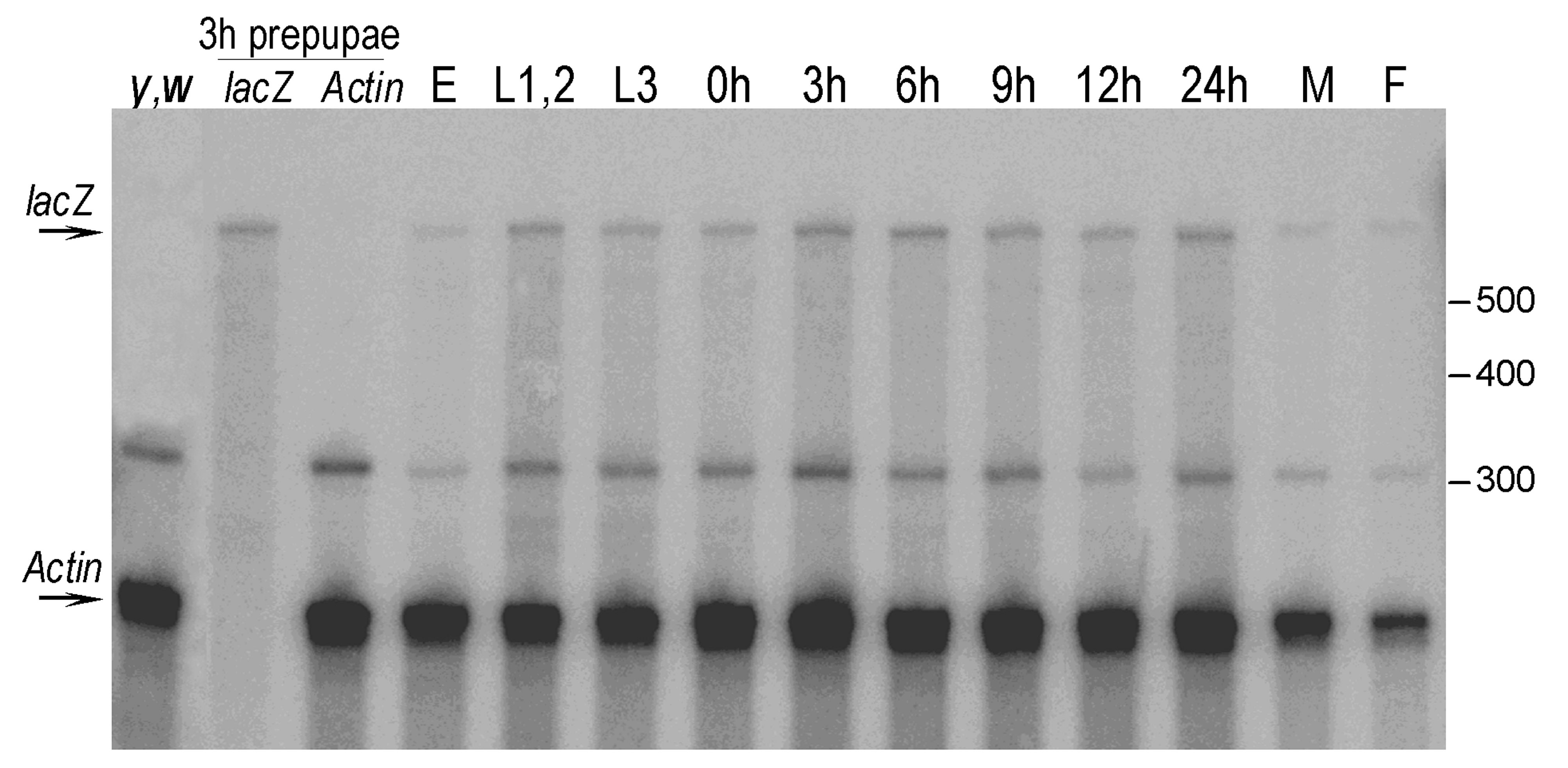

Supplement: Additional file 1 — BhC4-1-lacZ mRNA expression in an independent (-57/+40) transgenic line. Thirty micrograms of total RNA extracted from embryos (E), first and second instar larvae (L1,2), third instar larvae (L3), prepupae at 0 h, 3 h, 6 h, 9 h and 12 h, 24 h pupae, adult males (M) and adult females (F) were analyzed by the Ribonuclease Protection Assay using a mixture of two radiolabeled probes. The protected BhC4-1-lacZ RNA mRNA is 617 nt long (BhC4-1-lacZ arrow). The protected Actin mRNA is about 260 nt long (Actin arrow). In lane (y,w), 30 μg of total RNA extracted from embryos of the parental strain y,w were hybridized with both probes. In lanes (3 h prepupae/lacZ) and (3 h prepupae/Actin), 30 μg of total RNA extracted from 3 h prepupae of the (-57/+40) transgenic line were hybridized either with the lacZ probe or the Actin probe, as indicated. The migration of the RNA size markers is indicated on the right. [file 1471-2199-12-32-S1.TIFF]

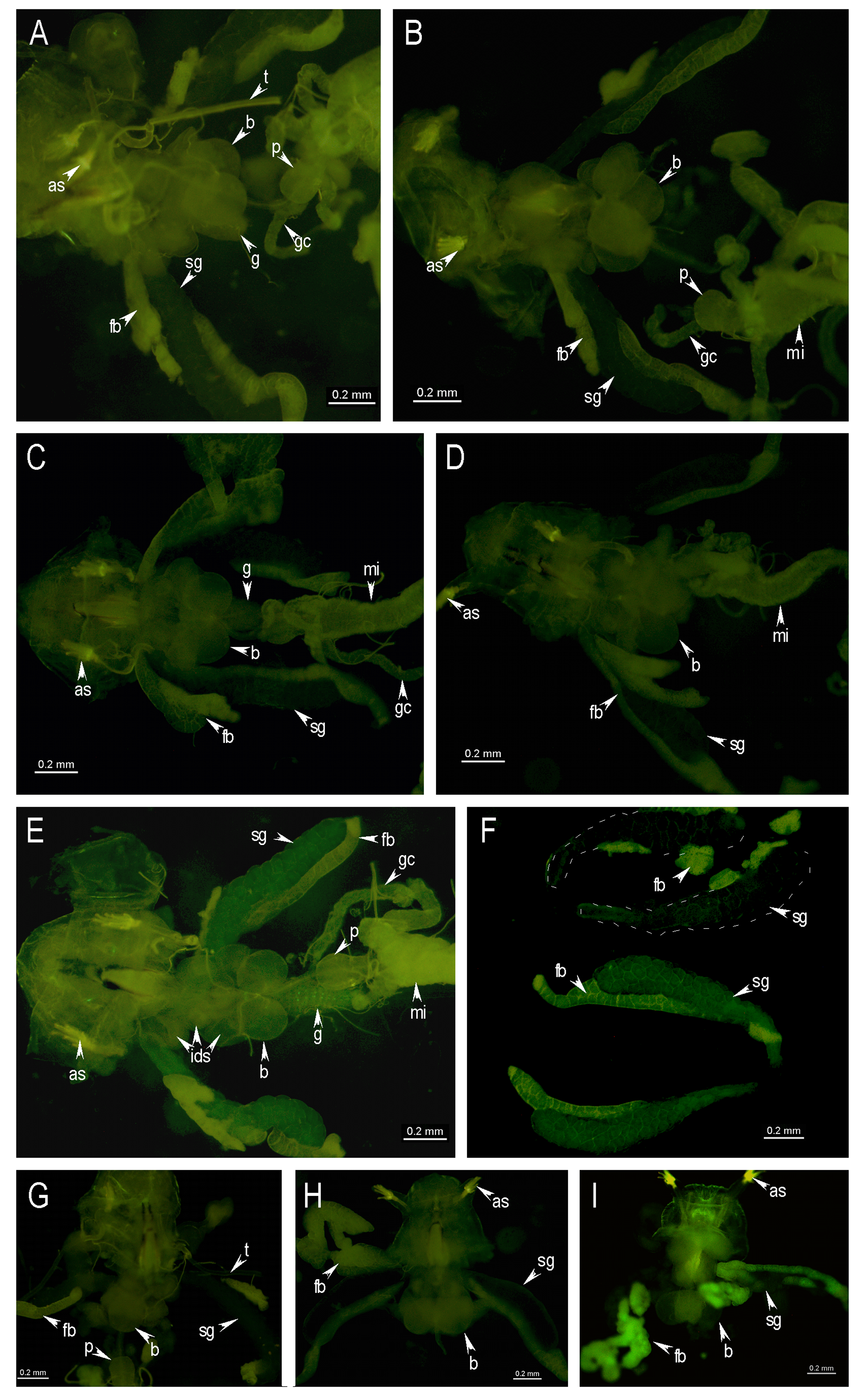

Supplement: Additional file 2 — Autofluorescence and GFP patterns of expression in control larvae. Third instar larvae were dissected and images were captured using a fluorescence stereoscope equipped with a GFP optical filter set. All images were captured with similar settings and exposure times. (A), (B) and (G) dissected third instar larvae of independent UAS-97 bp transgenic lines. (C) dissected third instar larva of the parental line, y,w. (D) and (H) dissected larvae of the Lsp2-GAL4 line. The larval tissues are identifiable due to the autofluorescence (yellowish) present in third instar larvae tissues. Note that some tissues (eg. fat body) present higher levels of autofluorescence when compared to others (eg. salivary glands). (E) dissected UAS-GFP.S65T third instar larvae. Note the occurrence of GFP expression in the salivary glands and in some groups of cells in the ventral ganglion (F) Salivary glands pairs were dissected either from a UAS-97 bp larva (top pair of salivary glands) or from a UAS-GFP.S65T larva (bottom pair of salivary glands). Both pairs of salivary glands were imaged together. Note the presence of GFP expression in the UAS-GFP.S65T salivary glands. The fat body associated to both pairs of salivary glands is identifiable due to the autofluorescence present in this tissue. The dashed white lines were drawn in order to indicate the location of the UAS-97 bp salivary glands in the image field. (I) Dissected UAS-97 bp; Lsp2-GAL4 larva. For comparison only the most anterior part of the larvae are shown in (G), (H) and (I). The larva shown in (I) was obtained after crossing the UAS-97 bp line (G), with the Lsp2-GAL4 line (H). GFP expression (bright green fluorescence) is only observed in the fat body of the UAS-97 bp; Lsp2-GAL4 larva (I). (as) anterior spiracle, (fb) fat body, (sg) salivary gland, (g) ventral ganglion, (gc) gastric caeca, (p) proventriculus, (b) brain, (t) trachea, (mi) midintestine, (ids) imaginal discs. Bar, 200 μm. [file 1471-2199-12-32-S2.TIFF]

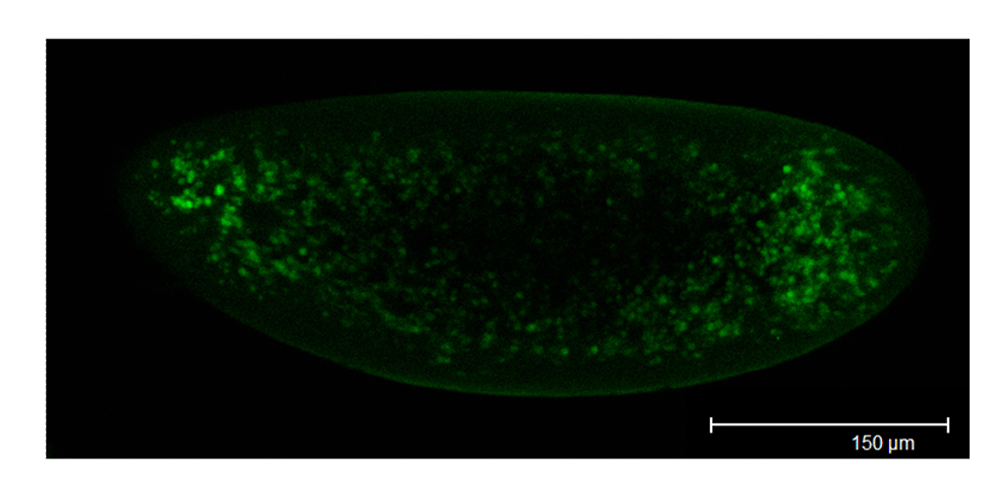

Supplement: Additional file 3 — Pattern of GFP expression in UAS-GFP.S65T/GAL4-twi.2xPE 3-4 h old embryo. Confocal microscopy of an UAS-GFP.S65T/GAL4-twi.2xPE 3-4 h old embryo. Note that the pattern of GFP expression is similar to the one obtained when the UAS-97 bp line was crossed to the same GAL4 driver line (shown in Figure 4A). [file 1471-2199-12-32-S3.TIFF]
